# Hypoxic link between cancer cells and the immune system: The role of adenosine and lactate

**DOI:** 10.32604/or.2025.065953

**Published:** 2025-07-18

**Authors:** EDUARDO ALVARADO-ORTIZ, MIGUEL ANGEL SARABIA-SáNCHEZ

**Affiliations:** 1 Programa de Posgrado en Ciencias Biológicas, Universidad Nacional Autónoma de México, Mexico City, 04510, México; 2 Departamento de Bioquímica, Facultad de Medicina, Universidad Nacional Autónoma de México, Mexico City, 04510, México; 3 Subdirección de Investigación Básica, Instituto Nacional de Cancerología, Secretaría de Salud, Mexico City, 14080, México

**Keywords:** Hypoxia, Hypoxia inducible factors (HIF-1/2/3α), Lactate, Adenosine, Immune evasion, Tumor microenvironment (TME)

## Abstract

The tumor microenvironment (TME) is characterized by a symbiosis between cancer cells and the immune cells. The scarcity of oxygen generates hostility that forces cancer cells to alter their biological features in solid tumors. In response to low oxygen availability, the Hypoxia Inducible Factors (HIF-1/2/3α) act as metabolic mediators, producing extracellular metabolites in the tumor microenvironment that influence the immune cells. The modulation of lactate and adenosine on immune evasion has been widely described; however, under hypoxic conditions, it has been barely addressed. Evidence has demonstrated an interplay between cancer and the immune cells, and the present review explores the findings that support HIFs bridging the gap between the rise of these metabolites and the immunosurveillance failure in a hypoxic context. Moreover, new insights based on systemic oxygen administration are discussed, which might counterbalance the effect mediated by lactate and adenosine, to recover anti-tumor immunity. Thus, the disruption of anti-tumor immunity has been the focus of recent research and this novel avenue opens therapeutic vulnerabilities that can be useful for cancer patients.

## Introduction

The TME (Tumor Microenvironment) is metabolically heterogeneous and commonly displays a differential oxygen gradient [1]. In this context, distinct metabolic sources are used for adaptation, maintenance, and progression under stressful conditions. The rapid proliferation of cancer cells, the less vascularized regions concerning blood vessels, and an abnormal blood vessel structure are causes of hypoxic sites, characterized by being at least 150 µm away from the vasculature, and with a pressure of less than 5 mmHg [2]. The low oxygen availability affects the metabolism of cells located in the hypoxic zone. Indeed, hypoxic TME is related to high extracellular levels of adenosine (up to 100 mM), lactate (up to 40 mM), and acidosis (pH less than 6.8) [3].

In the cancer field, two major avenues of study have emerged depending on the cell type of interest: cancer cells and non-cancerous cells residing in the tumor. The present review proposes an approach based on the metabolic changes hypoxic cancer cells undergo and how these affect surrounding cells, specifically immune cells, through extracellular metabolites. This approach complements the findings regarding how hypoxia directly triggers immune system evasion, a topic addressed in various previous reviews [4–6].

### The new insights into the Warburg effect

One century ago, Otto Warburg discovered that cancer cells maintain a fermentative pathway, in which glucose is metabolized to lactate, even under normoxic conditions. Warburg proposed that cancer cells drive a metabolic rewiring to maintain the metabolic fuels for cancer development. Although the so-called “Warburg effect” represents a less efficient mechanism in terms of ATP generation, the metabolic intermediaries necessary for anabolism are produced [7,8]. Initially, a mitochondrial disruption was the origin of cancer. The current viewpoints on the presence of oncogenes and the loss of tumor suppressor genes as the cause of cancer, with metabolic reprogramming being a consequence, not a cause.

For many years, the Warburg effect was attributed to cancer cells as a unique metabolic state, however, it seems that this does not necessarily occur. One reason is based on the functionality of mitochondria in subpopulations of cancer cells, due to this organelle being involved in metabolic processes, not limited to ATP production. Furthermore, a metabolic heterogeneity exists within the TME, with some cancer cells having a high glycolytic rate as a source of ATP, while others utilize Oxidative Phosphorylation (OXPHOS) or even a hybrid metabolism, depending on the cellular context [9].

The coexistence of oxidative and glycolytic cells in TME defines the metabolic state of cancer cells, as the waste products of one are the fuel of the other. Thus, multiple cell types within TME cause metabolic compartmentalization, triggering the “Reverse Warburg effect”. In this phenomenon, glycolysis occurs in non-cancerous cells, particularly in Cancer-Associated Fibroblasts (CAFs), which represent one of the most abundant non-malignant cells within solid tumors. The glycolysis-derived lactate is uploaded through Monocarboxylate Transporters (MCT) by the neighboring cancer cells, and employed to produce pyruvate, and subsequently, the synthesis of precursors for anabolism [10]. Glycolysis in CAFs is enhanced because OXPHOS decreases through mitophagy, as a consequence of reactive oxygen species in the TME [11]. Indeed, the release of lactate from CAFs and its incorporation into cancer cells support tumor malignancy [12]. Notably, the intercellular contact involved in the TME communication also led to the reverse Warburg effect [13]. Therefore, different cell types within TME, such as CAFs, transfer metabolites to meet the requirements of cancer cells and promote tumor progression. Because Hypoxia Inducible Factors (HIFs) act to mediate the synthesis, shuttling, and uptake of metabolites, the relationship between HIFs and metabolism is discussed.

### Extracellular metabolites and HIFs

The metabolic plasticity of cancer cells allows them to synthesize metabolic precursors that are necessary to adapt to stressful conditions, such as hypoxia [8,14]. The balance of glycolytic capacities and OXPHOS adjusted upon hypoxic conditions is widely recognized. Therefore, how can the Warburg effect and hypoxia be integrated? It has been argued that hypoxia encourages cancer cells to promote glycolysis, while the Warburg effect (aerobic glycolysis) seems to be favored in regions where oxygen is not a limiting factor [15,16]. Despite this, as mentioned above, metabolic heterogeneity is described in the TME, so how can a metabolic cancer profile be defined that explains the influence on other hallmarks, such as the immune system? One approach to addressing this issue is to consider factors, beyond metabolic enzymes, that emerge under low oxygen conditions, such as HIFs, which provide a complementary outlook. For instance, Glucose Transporter 1 (GLUT-1)-expressing cancer cells were found at the tumor edge, while HIF-1α was enriched in the tumor center [17].

HIF proteins are master transcription factors that regulate multiple responses related to stress conditions, through the formation of heterodimers between HIF-α and HIF-β subunits [18]. The function of HIF-α subunits depends on oxygen availability; hence, the transcriptional activity of HIFs is favored in hypoxia. Three HIF-α members have been described: HIF-1α, HIF-2α and HIF-3α. All of these proteins harbor an ODD (Oxygen-Dependent Degradation) domain that is targeted by PHDs (Prolyl Hydroxylase proteins) at proline sites, promoting the degradation of HIFs. The three HIF-α subunits induce gene expression by binding to HIF-β (also known as ARNT), which recognizes the Hypoxia Response Element (HRE) consensus sequence in target genes [16,19]. HIF-1α and HIF-2α have distinct functions depending on whether hypoxia is acute or chronic. HIF-1α seems to be active during the acute phase of hypoxia, while HIF-2α is responsible for adapting to chronic hypoxia [20]. The relevance of HIF-1α and HIF-2α has been explored in different types of solid tumors, but the function of HIF-3α is less well-known. Since HIF-1/2/3α synchronizes the hypoxic cellular adaptation, none of them should be excluded.

HIF subunits directly regulate the expression profile of metabolic enzymes. HIF-1α is mainly related to glycolytic response, while HIF-2α increases transcriptional responses associated with glutamine metabolism dependence or fatty acid synthesis [21–23]. Interestingly, the increased glycolytic rate induced by HIF-1α is improved under aerobic conditions, suggesting that the functions of HIF-1α are not limited to a hypoxic microenvironment [24]. Moreover, the mitochondrial activity in cellular adaptation to hypoxia was initially proposed as dispensable, but actually, it is well-known that mitochondria are employed for glutaminolysis and acetate metabolism, suggesting the recovery of additional functions through anaplerotic reactions. In the cases of mitochondria as an energy source, ATP reaches the TME. The extracellular ATP establishes a feedback loop that sustains HIF-1α activity, enhancing a transcriptional response related to chemoresistance [25]. Strikingly, extracellular ATP in TME originates from various sources, including cell death rates during tumor progression, ongoing exposure to chemotherapeutic drugs, release via specific transporters, and ATP released from extracellular vesicles [26,27]. Metabolic enzymes act on extracellular ATP, leading to the formation of adenosine, which can then act on designated receptors, producing an immunosuppressive state. Notably, different cell types within the TME can express enzymes that promote the accumulation of extracellular adenosine [28].

**Figure 1:**
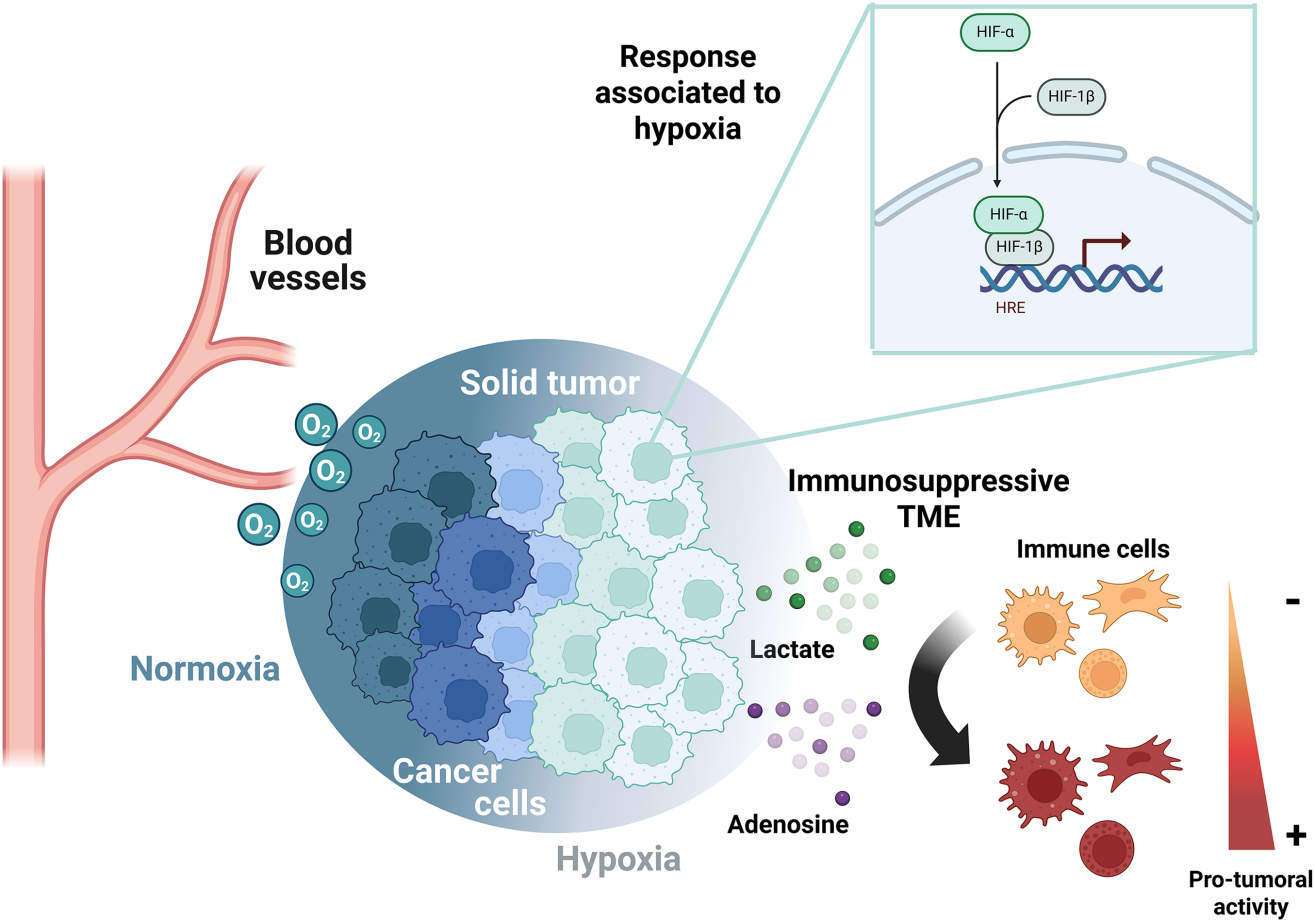
Metabolic reprogramming in cancer cells determines immunosuppression in the TME. The oxygen gradient established in solid tumors upregulates HIF-α subunits in hypoxic areas, which restricts the immune system through the release of lactate and adenosine. HRE, Hypoxia response element; TME, tumor microenvironment. The figure was created with BioRender.com

Target genes of HIF-1α are related to tumor progression, affecting proliferation, angiogenesis, and metastasis [16]. Also, the tumoral hypoxic zones are described as immune-privileged niches [29]. The extracellular factors modulated by hypoxia and HIF subunits that negatively regulate the immune cells include the overexpression of Vascular Endothelial Growth Factor (VEGF), externalization of phosphatidylserine to the outer membrane, and accumulation of metabolites (adenosine and lactate) within the TME [30]. This is achieved due to the upregulation of the transcriptional activity of HIFs by the low oxygen availability, which indirectly enriches adenosine and lactate in the TME, supporting immune evasion, as will be explained in detail in the review (Fig. 1).

Due to the metabolic reprogramming in cancer, lactate and adenosine coexist in the TME, inducing a plethora of functions in the surrounding cells. Therefore, the metabolic enzymes and transporters associated with these metabolites may be crucial to understanding the effects on immune cells.

## Adenosine in a Hypoxic Microenvironment

Extracellular ATP accumulates in normal tissues at concentrations ranging from 10 to 100 nM. In tumor tissues, this concentration increases significantly, and extracellular ATP is metabolized by CD39/CD73 ectonucleotidases [31]. Adenosine is synthesized through several enzymatic steps. First, CD39 generates ADP and AMP from ATP. Then, CD73 converts the accumulated AMP into adenosine. Thus, extracellular adenosine in solid tumors primarily originates from extracellular ATP rather than from direct adenosine release [28].

The dynamism of tissue oxygen concentration requires mechanisms that maintain membrane permeability in different physiological processes. Once adenosine is generated, it maintains a basal concentration of around 200 nM. However, if damage occurs, the concentration can reach up to 100 μM [32]. Adenosine release occurs at the same time as a reduction in adenosine-metabolizing enzymes [33]. If exposure to chronic hypoxia occurs, it disrupts the blood flow to defined regions, activating ischemia-related processes. Adenosine is well-documented for protecting against damage induced by hypoxia; conversely, the absence of adenosine promotes inflammatory infiltration. Thus, adenosine formation can potentially act as an immune cell regulator within hypoxic sites, modulating vascular permeability [34,35]. Moreover, adenosinergic receptors inhibit pro-inflammatory responses, indicating that adenosine is an important protector against hypoxic damage [36].

**Figure 2:**
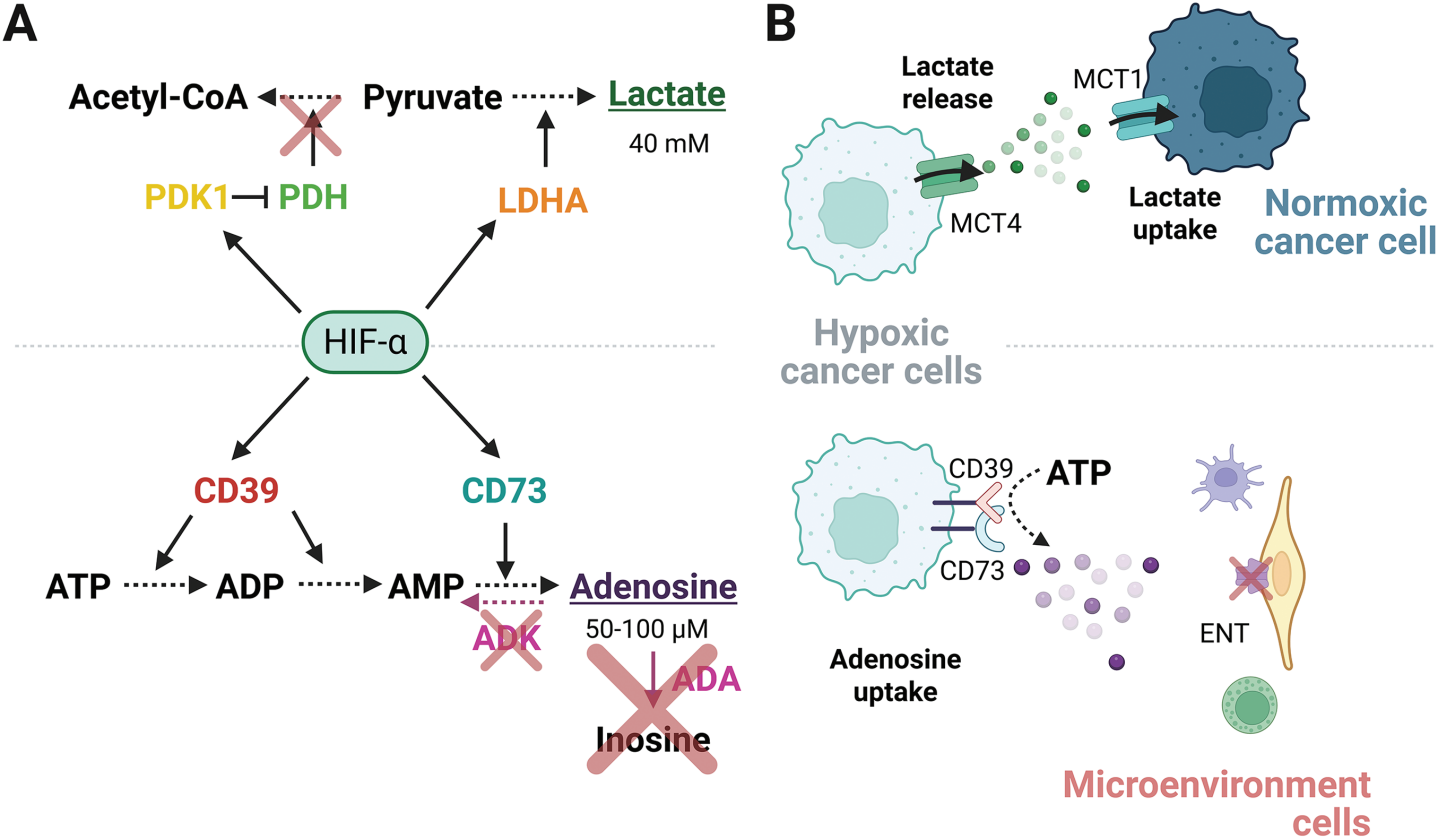
Regulation of high levels of lactate and adenosine in the TME. (A) HIF-α subunits transcriptionally upregulate metabolic enzymes that elevate the concentration of lactate and adenosine. (B) Extracellular lactate secreted by hypoxic cancer cells is taken by normoxic cancer cells to use as an energy source, establishing a metabolic symbiosis. Conversely, the downregulation of nucleoside transporters in non-malignant cells prevents the extracellular adenosine uptake released by hypoxic cancer cells. ENT, Equilibrative nucleoside transporters; TME, tumor microenvironment. Figure was created with BioRender.com

Importantly, HIF-1α regulates the expression of CD39/CD73 ectonucleotidases during hypoxia [37]. Indeed, the promoter region of CD39/CD73 contains an HRE in the human genome. This was experimentally demonstrated in mice, where liver failure induced HIF-1α activity and expression of CD39/CD73 [38]. Accordingly, previous reports showed that CD39 and CD73 are regulated by HIF-1α and identified their binding site in the promoter region [37,39], reinforcing the premise that HIFs are capable of mediating extracellular adenosine accumulation. In this manner, HIFα modulates the adenosine levels, upregulating the expression of CD39 and CD73, which participate in the conversion of ATP to adenosine (Fig. 2A). Although this mechanism is less explored for the other HIFs, the blockade of HIF-2α disrupts the CD73 expression and the extracellular adenosine accumulation in glioblastoma cells [40]. Furthermore, in triple-negative breast cancer cells, both HIF-1α and HIF-2α can bind to the promoter region of CD73 and other genes encoding immunosuppressive molecules, such as PD-L1. The evidence suggests that both HIFα subunits can potentially maintain an immunosuppressive state [41]. Currently, the influence of HIF-2α and HIF-3α subunits on the adenosinergic response remains unclear.

Extracellular adenosine is highly unstable, influenced by enzymes that modulate its lifetime through its conversion to other products. For instance, Adenosine Deaminase (ADA) favors the adenosine conversion to inosine, meanwhile, Adenosine kinase (ADK) converts adenosine to AMP through its phosphorylation. The activity of ADA and ADK maintains a compartmentalized concentration of adenosine, coordinating the influence at the extracellular space, or regulating its uptake through specific transporters [42]. The Equilibrative Nucleoside Transporters 1-4 (ENT1-4) belong to a group of proteins involved in mediating the uptake or release of adenosine in a concentration-dependent manner. Hypoxia maintains extracellular adenosine levels by restricting its intracellular uptake or enhancing its release, in a cell-dependent manner. Hypoxia downregulates ENT1, which decreases adenosine uptake in umbilical endothelial cells [43]. Therefore, TME cells not only generate adenosine but are also able to capture it, establishing a recycling mechanism to produce energy in the cells that use it. Simultaneously, the responsiveness of non-cancerous cells and non-immune cells decreases, allowing the metabolite to act preferentially on cancer and immune cells (Fig. 2B).

In different types of tissues, hypoxia exposure decreases ADK activity by transcriptional repression mediated by HIF-1α. This acts as a mechanism to diminish vascular leakage and ischemic tissue damage [44]. However, how is the repressive nature of HIF-1α explained? This question was explored in the context of cancer, where hypoxia-induced ADK repression maintains the extracellular adenosine concentration necessary to maintain an immunosuppressive microenvironment. Increased activity of the MXI1 repressor, in a HIF1α mechanism-dependent manner, was involved in this process. Because MXI1 acts upstream of the promoter region of ADK, this mechanism explains why adenosine is not metabolized in liver cancer cells [28]. Together, these results highlight adenosine as an important driver of malignancy, where different enzymatic steps promote its synthesis and prevent its metabolism. Adenosine functions are crucial to tumor malignancy and extend to the TME. Treatments aimed at regulating adenosine concentration have been explored. For example, recombinant ADA reduces the proliferative capacity of cancer cells and inhibits cellular migration induced by hypoxia, through HIF2α degradation [45].

Furthermore, adenosine receptors are ubiquitously expressed in different cell types, including cancer and non-cancer cells. Thus, the effect of adenosine on the TME is not limited to a single cell type. In this sense, adenosine enhances malignancy through adenosine receptors. For instance, the A2B receptor maintains the “stemness” properties, including tumor initiation capacity and chemoresistance. Additionally, adenosine promotes proliferation, EMT, invasion, and metastasis in gastric cancer, in an A2A-dependent process [46]. Thus, adenosine receptors are required to mediate malignancy. The functional effects of adenosine receptors on cancer and immune cells have been further explored by other authors [47].

## Lactate in a Hypoxic Microenvironment

In non-malignant cells, the glucose is metabolized to pyruvate through glycolysis. Pyruvate is then oxidized through the TCA cycle to generate the reductive power necessary for OXPHOS. This process produces ∼36 molecules of ATP per glucose molecule catabolized [7]. In contrast, cancer cells catalyze the conversion of pyruvate to lactate via the enzyme Lactate Dehydrogenase A (LDHA), regardless of oxygen availability. However, during hypoxia, the conversion of glycolysis-derived pyruvate to lactate rather than to acetyl-CoA is favored. This is achieved because HIF-1α promotes the expression of LDHA and Pyruvate Dehydrogenase Kinase 1 (PDK1), an enzyme responsible for inhibiting Pyruvate Dehydrogenase (PDH) [48]. The upregulation of PDK1 prevents the oxidation of pyruvate to acetyl-CoA, and it contributes to maintaining the lactate fermentation (Fig. 2A) [49]. In solid tumors, the overall levels of acetyl-CoA are diminished, as is the TCA cycle and OXPHOS, when oxygen is restricted, reducing the supply of ATP through OXPHOS [50]. Notably, the transcription targets regulated by HIF-1α are consistent with the pro-Warburg effect observed for this subunit, and the main reason for the enrichment of extracellular lactate is the upregulation of LDHA due to improved glycolysis (Warburg effect).

Glutaminolysis also contributes to elevated lactate levels because uptake by the ASCT2 transporter and conversion to α-ketoglutarate allow entry into the TCA cycle. Then, malate is metabolized to pyruvate by the malic enzyme and converted to lactate via LDHA. Of note, malic enzymes and LDHA are overexpressed in tumors [51].

Studies have shown that lactate is heterogeneously distributed in tumors, reaching concentrations of up to 40 mM [52]. The unequal distribution of lactate within the tumor originates from the differential metabolic profiles but is also associated with oxygen availability, as mentioned above. Hypoxic cancer cells favor glucose uptake by upregulating the glucose transporter GLUT-1, thereby increasing glycolysis and, therefore, lactate release. The secreted lactate is then taken up by normoxic cancer cells and converted to pyruvate by LDH-B. In consequence, pyruvate enters the TCA cycle and is used by the mitochondria to produce energy. Hence, lactate is produced in an oxygen-poor zone and utilized in an oxygen-rich zone [53,54]. Additionally, the lactate contributes to the survival of cancer cells located far from the blood vessels [16]. The amount of lactate in the extracellular space depends not only on the metabolic route that produces it but also on the transporters responsible for its mobilization. Specifically, MCT4 mainly drives the lactate release under hypoxic conditions, in contrast to the lactate capture by MCT1 to internalize it into the cells [55,56]. Notably, the export of lactate out of the cell is necessary for oncogenesis, since the inhibition of MCT4 triggers the accumulation of intracellular lactic acid and the impaired survival of the hypoxic cancer cells [57]. Altogether, the hypoxic cancer cells release lactate through MCT4, and subsequently, after diffusion along the TME, lactate is internalized through MCT1 within the normoxic cancer cells. This phenomenon is widely known as metabolic symbiosis, where different cell types obtain a mutual benefit (Fig. 2B).

## The Hypoxia-Adenosine-Immune Evasion Network

**Figure 3:**
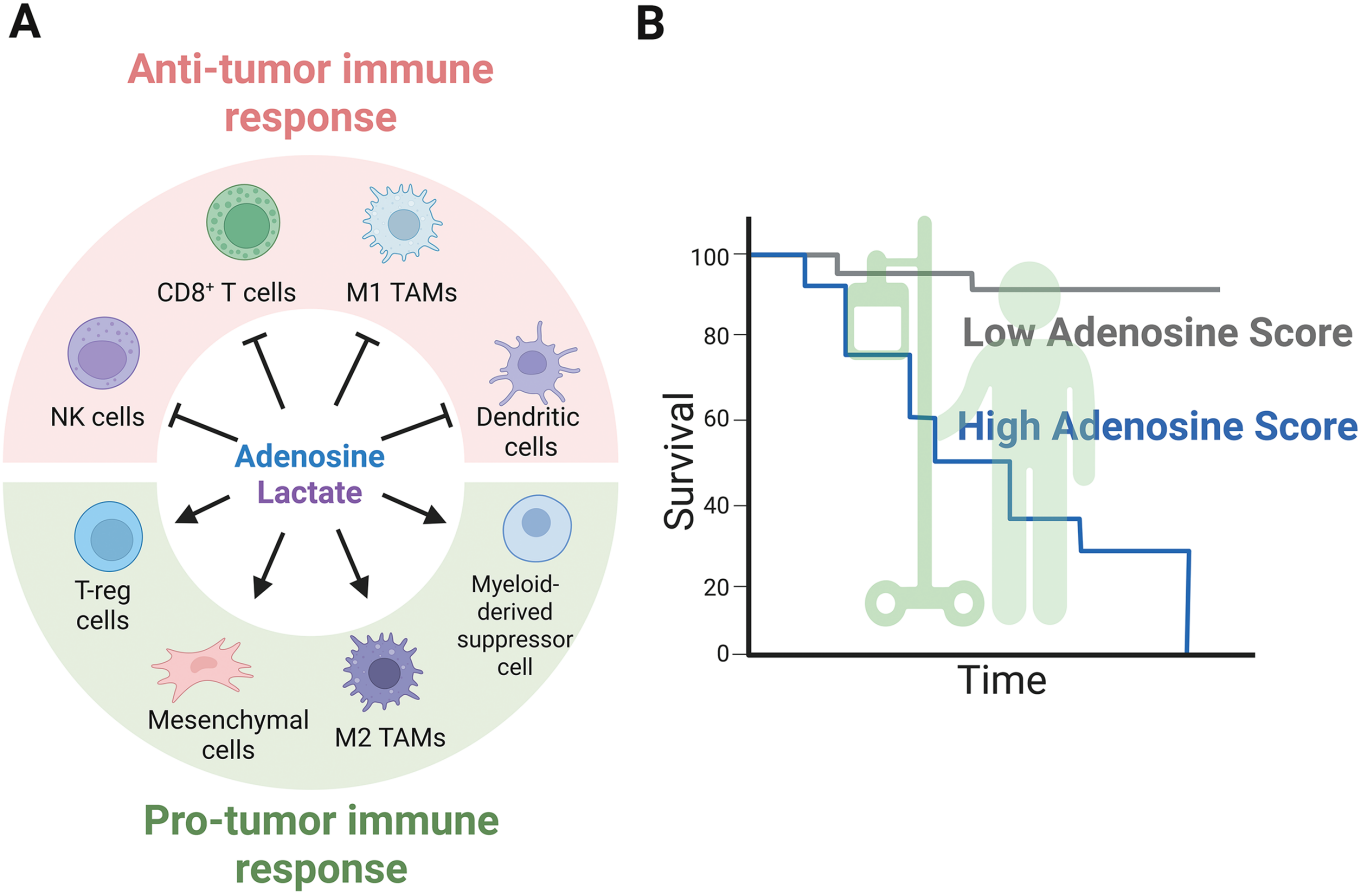
Extracellular metabolites mediate anti- and pro-tumor immune responses. (A) Extracellular metabolites such as adenosine and lactate suppress anti-tumor immunity, as illustrated by the actions of NK cells, CD8^+^ cells, M1 macrophages, and dendritic cells, while promoting the infiltration of T-reg cells, M2 macrophages, mesenchymal stem cells, and myeloid-derived suppressor cells, all of which possess pro-tumor capabilities. (B) A schematic representation of cancer patient survival linked to high and low adenosinergic signatures explored across various solid tumor types. Figure was created with BioRender.com

The immunosuppressive microenvironment is achieved by adenosine through mechanisms influenced by hypoxia (Fig. 3A). Identifying adenosine in solid tumors is a challenge that was recently explored. Employing mass spectrometry, researchers found that extracellular adenosine is ubiquitously distributed in pancreatic adenocarcinoma, particularly in hypoxic regions where HIF1α is present [58]. Adenosine affects the function of different cell types within the TME, including regulatory T-lymphocytes (T-reg), cytotoxic T-lymphocytes, NK cells, and macrophages. Moreover, the CD39/CD73 ectonucleotidases are expressed in immune system cells. Recently, NK cells expressing CD73 with the capacity to reduce the CD4^+^ T-lymphocyte population have been observed in breast cancer, suggesting increased extracellular adenosine [59]. However, this population does not synthesize adenosine, suggesting that additional mechanisms may be involved in the immunosuppressive capacities independently of ectonucleotidase activity. In addition, CD73 expression is intrinsically associated with poor clinical outcomes. Thus, adenosine production can act on CD8^+^ lymphocytes, causing anergy [60]. Furthermore, mesenchymal cells upregulate CD39 to maintain adenosine synthesis, a process complemented by CD73 expression on T-lymphocytes, while simultaneously decreasing ADA activity [61]. This effect is crucial for reducing the activation of T-lymphocytes, suggesting that the immunosuppressive properties of adenosine are influenced by a network of multiple cell types. Additionally, several reports indicate that hypoxia can affect the paracrine functions of mesenchymal cells, promoting angiogenesis and preventing apoptosis as a protective mechanism in response to damage. However, the role of adenosine in enhancing the malignant properties of cancer cells is not well understood [62,63].

Adenosine generated by a hypoxic microenvironment promotes the enrichment of specific plasmacytoid Dendritic Cells (pDCs). In hepatocellular carcinoma, pDCs are highly infiltrated and play a central role in promoting immunosuppression. Adenosine acts on pDCs, modifying immune cells by increasing T-reg cells and decreasing CD8-mediated cytolysis [64]. What is the interplay between adenosine, hypoxia, and immune evasion? As described earlier, CD39 expression is directly associated with HIF1α activity in liver cancer, maintaining the enzymatic conversion of ATP to AMP. This mechanism increases myeloid-derived suppressor cells and prevents their differentiation into dendritic cells. Thus, the immune system suppression is maintained while cytotoxic T-cell responses are downregulated and the T-reg population is augmented, thereby diminishing the anti-tumor immune response. Moreover, the A2A receptor is responsible for this immunosuppressive state; therefore, the use of an adenosine receptor antagonist together with immunotherapy (Anti-PD1) could be a promising clinical approach [28,39,65]. Similar data reports that CD73 expression is intrinsically associated with poor clinical outcomes. In this case, adenosine production acts on CD8^+^ lymphocytes, causing energy [60]. Furthermore, HIF1α induces simultaneous increased expression of CD73, CD47, and PD-L1 in triple-negative breast cancer [41]. This evidence suggests a positive feedback loop in which HIF1α mediates adenosine production to increase immunosuppression. Additionally, blocking CD73 combined with anti-PD1 immunotherapy promotes CD8^+^ tumor infiltration and decreases tumor cell growth in the colorectal cancer model [66]. Therefore, the influence of HIFα subunits on the adenosinergic pathway in different cell types of the TME is a promising area of research.

Macrophages are another cell type influenced by adenosine. There exist two types of polarization: anti-tumoral M1 macrophages and protumoral M2 macrophages. M2 macrophages are predominantly found in hypoxic regions, where adenosine is most abundant. The M2 macrophages express the A2A adenosine receptor; therefore, blocking the adenosinergic pathway affects M2 macrophage infiltration into solid tumors, as well as the expression of the PD-L1 immune checkpoint protein. Importantly, the adenosinergic signature in this condition indicates a poor prognosis and survival rate for pancreatic cancer patients (Fig. 3B) [58].

T-reg cells can reduce the exacerbated CD8^+^ and CD4^+^ lymphocyte function, thereby suppressing anti-tumor immunity in the presence of adenosine and hypoxia [65]. However, the study of both factors is poorly explored. Hypoxic solid tumors show higher T-reg cell infiltration, as previously demonstrated in breast cancer [67]. Similarly, hypoxia induction in a hepatocellular carcinoma model increases T-reg infiltration, which is strongly associated with poor clinical outcomes [68]. In gastric cancer, adenosine synthesized in the TME acts on T-reg cells to suppress the cytolytic capacities of CD8^+^ cells and promote their proliferation [69]. Thus, although the individual effects of adenosine and hypoxia on T-reg cells have been explored, both undoubtedly mediate the immunosuppressive characteristics of the TME.

## The Hypoxia-Lactate-Immune Evasion Network

The idea that cancer cells produce lactate for export rather than use it as a nutrient has been discussed [56]. Therefore, lactate is expected to act as a signal to nearby cells, both cancerous and non-cancerous. The effect of lactate on immune system cells has been a recurrent field of exploration from the perspective of pH regulation [70]. The acidification leads to impairment of anti-tumoral immune cells, for example, modulating the function of NKT and NK, or the recruitment of T-reg cells [71,72]. Lactate release and extracellular acidification are increased in co-cultures of cancer cells and Tumor-Associated Macrophages (TAM), demonstrating the dependence of metabolic profiles on intercellular communication [73]. It should also be taken into account that the pH is modulated by other components, such as proton pumps, which positively regulate the activity of macrophages [74]. Moreover, extracellular acidification was predominantly demonstrated in hypoxia rather than in normoxia, in a glucose-independent manner [75]. Accordingly, glycolysis-deficient cells were able to acidify the extracellular environment [56]. Microenvironment acidification and low oxygen concentration are two important criteria that can influence the immune system cells, and although both are interrelated in the TME, it has been observed that the TAMs are mainly located in hypoxic zones [75].

As mentioned above, lactate is produced in both the absence and presence of oxygen; therefore, it is risky to assume that lactate plays a similar biological role in both conditions. Regulation of lactate on the immune cells has been extensively detailed, but there are few cases where it is considered to occur in a hypoxic context. For instance, a cancer cell-conditioned medium induced M2 macrophage polarization in a normoxic environment, but this effect was amplified when both cancer cells and macrophages were exposed to hypoxia and a higher glucose concentration [75]. Metabolomic screening demonstrated that, while lactate positively correlates with M2 polarization under hypoxic conditions, the metabolic product 2-amino-butanoic acid (2A-BA) correlated with M2 polarization under normoxia [75].

The cytokines and lactate generated by TAMs and cancer cells, respectively, establish a positive feed-forward loop that promotes tumor progression [76]. The pleiotropic functions of lactate and Extracellular Vesicles (EVs) are also implicated in intercellular communication between cancer cells and the immune cells. Among the various components shown to be transported within EVs, long non-coding RNAs (lncRNAs) have recently attracted attention. TAMs release HIF-1α-stabilizing long noncoding RNA (HISLA), which is contained in EVs. HISLA restricts the binding of PHD2 with HIF-1α, thereby downregulating the hydroxylation and degradation of HIF-1α. This is a non-cancer cell-mediated mechanism by which HIF-1α levels are regulated in cancer cells. Notably, the cancer cell, in turn, produces lactate, which augments HISLA in macrophages, establishing a feed-forward loop. Lactate release is due to metabolic reprogramming caused by HISLA blockade, which impairs glycolysis in cancer cells *in vivo* [73]. Why is this relevant? The Warburg effect refers to glycolysis occurring in the presence of oxygen. Although it may seem counterintuitive that HIFs participate in this phenomenon, evidence from the interaction between the macrophages and cancer cells suggests a potential role for HIFs in tumor areas beyond hypoxic ones. Notably, the HISLA lncRNA is also expressed in B-lymphocytes, hence, the involvement of additional immune cells in EV-mediated lactate production in cancer cells seems possible.

Importantly, macrophages respond to hypoxic conditions by generating lactate due to their increased glycolytic rate, which mediates histone acetylation and affects the expression of target genes involved in macrophage polarization. This function reveals that lactate has implications for gene expression and favors tumor progression through epigenetic mechanisms [77]. Notably, macrophages have been shown to express HIF-1α, which requires LDHA activity in cancer cells. This emphasizes the role of lactate and intercellular communication, although it remains to be defined whether lactylation is involved [78]. M2-associated genes are regulated by HIF-1α [79], so both lactate and HIFs regulate macrophage plasticity, activating or restricting the immune response. However, M2 polarization was associated with the loss of HIF-1α and overexpression of HIF-2α [80]. Therefore, the explanation for these contrasting results remains to be elucidated.

TAMs have been found to infiltrate hypoxic areas with a tendency towards immune escape [81]. Additionally, TAMs harboring an M2 phenotype are mainly located in the hypoxic areas of solid tumors [82]. So, then, do HIF subunits mediate macrophage polarization? Interestingly, HIF-1α-deficient macrophages were recruited to infiltrate hypoxic regions at rates similar to those of macrophages harboring the wild-type version of HIF-1α. However, HIF-1α-deficient macrophages exhibited enrichment of M2 phenotype markers and reduced cytotoxicity to tumor cells, suggesting a relationship between HIF subunits and macrophage polarization [80]. Furthermore, HIF-1α can regulate immune cell infiltration into the tumor. For example, the expression of THBS2, a calcium-associated glycoprotein, was associated with response to immunotherapy. Higher THBS2 expression responded to CTLA4, while lower THBS2 expression showed less immune cell infiltration, but indicated better patient response to anti-PD-L1 therapy. Remarkably, upregulation of THBS2 favored anaerobic metabolism, enhancing lactate secretion. This enrichment of extracellular lactate was reversed when HIF-1α was downregulated [83]. One of the main mechanisms for lactate action is through GPR132 to modify macrophage polarization [84]. However, infiltration of cytotoxic T-lymphocytes, helper T-lymphocytes, and T-reg cells, which are favored by the overexpression of THBS2, was ablated in mice lacking GPR132. This suggests that THBS2 has a global function that partially depends on the HIF-1α subunit [83].

It is widely known that cancer cells release a variety of immunosuppressive molecules [85]. In the case of lactate, rather than being a toxic byproduct, it is utilized by the immune cells as part of the adaptive response in hypoxic conditions, but what are the implications of lactate release for immunotherapy? High levels of lactate and hypoxia impair the cytotoxicity mediated by T lymphocytes and modulate T-reg cells [86]. Notably, T-reg cells take up lactate and induce the PD-1 expression to achieve immunosuppression [86], so the function of lactate in immune checkpoints might be critical for the effectiveness of treatments targeting PD-1.

## Adenosine and Lactate: Two Birds in the Same Nest

Adenosine and lactate are enriched in response to hypoxia, however, research aimed at understanding the implications of both metabolites combined is limited. An example of the interconnected role between both metabolites in the same cellular context has been described in cancer cells. Lactate increased the expression of CD38, which is involved in the conversion of NAD^+^ to adenosine, through a CD39/CD73-independent mechanism. In this case, the adenosine contributed to invasion and metastasis [87]. Furthermore, the adenosine-producing enzymes CD39/CD73 have been directly associated with the expression of LDH5 and HIF-1α [88]. In this same report, CD39 in CAFs was related to the expression of PD-L1 and PD-1 on cancer cells and tumor-infiltrating lymphocytes, respectively [88]. These findings recognize that deepening our knowledge of metabolism in the effectiveness of immunotherapy involves considering both cancer cells and the non-cancerous cells that surround them.

In addition to acting as extracellular metabolites, lactate and adenosine regulate epigenetic mechanisms in cancer. The N6-methylation of Adenosine (m^6^A) modulates splicing, stability, and translation of the target RNAs. On the other hand, lactate is involved in histone acetylation to regulate gene transcription. Notably, the lactate-mediated lactylation affects m^6^A modifications to affect the stability of transcripts [89]. Of note, the transcript of lactate transporter MCT1 was described as one of the m^6^A targets, and this regulation enhanced the uptake of lactate to impair cytotoxic CD8^+^ lymphocytes [90].

The restoration of the immune cells by immunotherapy leads to immune-related adverse events in some patients. Both adenosine and lactate have been associated with these events in gastrointestinal cancer patients, postulating them as possible prognostic markers for the application of the Immune checkpoint inhibitor [91]. Advances in diagnosis have been made in other diseases. For instance, the measure of LDH and ADA in the pleural fluid has shown an accurate diagnosis of tuberculous pleural effusion [92]. Regarding cancer, the presence of both metabolites in serum has been evaluated as a diagnostic tool in colorectal cancer; however, they exhibited different features. While lactate was useful for contrasting colorectal adenoma with normal tissue, adenosine distinguished between colorectal adenoma and colorectal cancer [93].

As explained, HIFs modulate the amount of lactate and adenosine by acting upstream as a transcriptional regulator, regulating metabolic enzymes. Therefore, by inhibiting the HIF-1α subunit, the presence of adenosine and lactate is reduced [94]. Due to HIF-1α blockade abrogating the expression of additional immune system regulators, a function as an immunoadjuvant was considered [94]. Hence, elucidating how adenosine and lactate interplay to control the immune system will undoubtedly provide new therapeutic approaches.

## Searching for Therapeutic Options: The Answer Is in the Air

**Figure 4:**
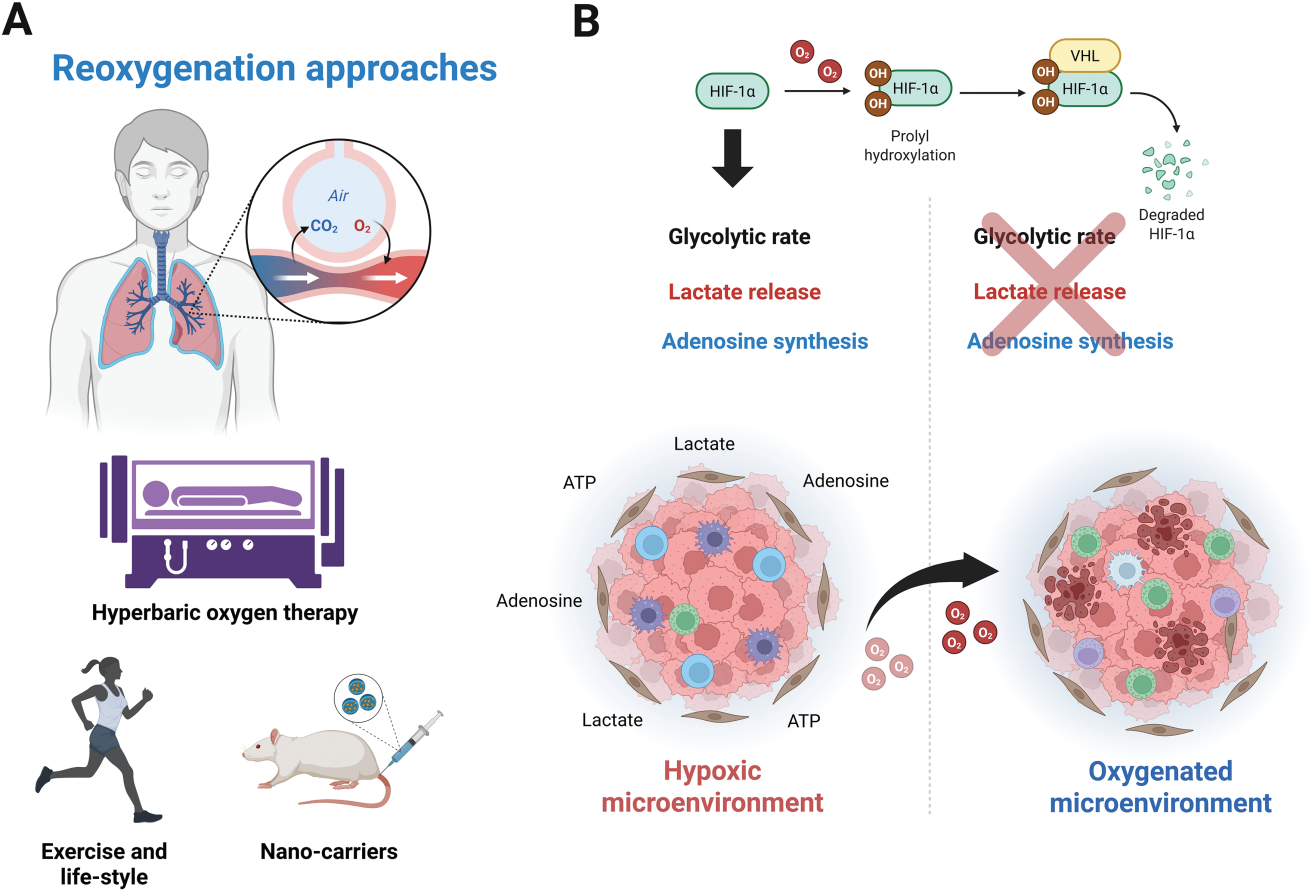
Therapeutic approaches focused on increasing oxygen infiltration. (A) Oxygen-restoring strategies based on breathing capacity above atmospheric levels explored *in vivo*, such as hyperbaric oxygen therapy, exercise, lifestyle, or nano-carriers. (B) Tumor infiltration and oxygen-dependent mechanisms are downregulated by recovering oxygen in the TME. Figure was created with BioRender.com

Hypoxia is strongly linked to the disruption of immunosurveillance due to increased adenosine and lactate concentrations. How can this knowledge be useful in therapy? Cells must adapt to a hostile microenvironment to access hypoxic conditions. Recently, hypoxia-modified Chimeric Antigen Receptor (CAR) T-cells have been proposed as a tool to address this issue, by taking advantage of this condition [95]. However, other researchers suggest that oxygen levels can be modulated *in vivo*. Evidence shows that oxygen recovery can restore anti-tumor immunity. To this end, various strategies have been developed, such as nano shuttles or liposomes containing catalase and H_2_O_2_ [96]. Despite substantial evidence supporting the possibility of modulating the oxygen supply, many methods remain methodologically and economically unfeasible. However, the solution may be simpler, as exercise, the lifestyle choices of cancer patients, as well as hyperbaric therapy, could significantly influence the endogenous anti-tumoral response by regulating oxygen concentrations in solid tumors (Fig. 4A).

In mice, hyperoxic breathing at 60% O_2_ decreases the immunosuppression linked to adenosine release, reduces A2A expression, and promotes the recruitment of anti-tumor immune cells, in contrast to breathing at 21% O_2_. Importantly, while no data is available for cancer patients, in healthy individuals, the reduction in plasma adenosine concentration during exercise is comparable to that achieved when subjects are exposed to a hyperbaric environment (Fig. 4A) [97,98].

Similarly, the ability to target new antigens through machinery associated with Major Histocompatibility Complex (MHC-I) depends on oxygen levels and HIF-1α activity. Since HIF-1α acts as a repressor of the MHC-I complex, it has been suggested that when oxygen concentration gradually increases to atmospheric levels (21% O_2_) or even exceeds hyperoxic conditions (60% O_2_), the capacity for antigen presentation is significantly enhanced [98]. Additionally, hyperoxic exposure reduces components related to the adenosinergic pathway in solid tumors, including extracellular adenosine, CD39/CD73 ectonucleotidases, and adenosine receptors (A2A, A2B). Furthermore, the ability to present antigens through MHC-I was restored, and NK cell-mediated cytolysis was improved, thus restricting tumorigenesis and the metastatic capacities of cancer cells [99].

For lactate, although the enrichment of intracellular lactate and the acidification of cancer cells were also observed under 60% oxygen exposure, hyperoxia exposure restrained the tumor growth and metastasis. This phenomenon is attributable to the downregulation of lactate export mediated by MCT1. Hyperoxia and deletion of MCT1 impair glycolysis; however, the function of HIF subunits was not evaluated [100]. Similarly, HIF-1α is downregulated by hyperbaric oxygen treatment in a manner consistent with degradation mediated by O_2_ availability, which decreases glycolytic flux under low and high glucose conditions. Hyperoxia suppresses the Warburg effect due to the downregulation of key genes involved in glycogen metabolism and glycolysis in a HIF-1α-dependent manner. Moreover, this report showed that hyperbaric oxygen therapy reverted the production of pyruvate and lactate by inhibiting the HIF-1α/Phosphofructokinase Platelet (PFKP) axis [101]. In addition, hyperbaric oxygen therapy reversed LDH activity elicited by hypoxia [102]. Notably, measurements of metabolites in blood samples under hyperoxia-stimulated conditions revealed decreased pyruvate efflux with no effect on PDH activity, highlighting the complexities of this physiological state that require further investigation [103].

The success of therapy depends on the ability to access the hostile environment of hypoxic tumors, which are characterized by high deposition of extracellular matrix. This condition affects the availability of conventional drugs and antibodies used in immunotherapy to act on their targets. For example, in a pancreatic orthotopic mouse model, the combined use of conventional therapies, Abraxane and gemcitabine, along with oxygen recovery, decreases the tumor cell growth by re-establishing the anti-tumoral immune response, reducing the deposition of extracellular matrix, and increasing T-lymphocytes infiltration, as well as the M1/M2 macrophage ratio [104,105]. Similar effects have been observed in other types of solid tumors, in which hyperbaric therapy is effective at eradicating functional properties associated with stemness, enhancing drug uptake within tumors, reducing vascularization, and decreasing the metastatic potential of cancer cells [105]. Despite the benefits of hyperbaric therapy, this approach may be prone to complications. For example, mice treated with doxorubicin are at risk for cardiomyocyte damage. Because systemic exposure to high concentrations of oxygen can also contribute to this dysfunction, as indicated by elevated serum creatine phosphokinase activity, chemotherapy-induced cardiomyopathy may be slightly exacerbated by hyperbaric oxygen therapy. This has prompted researchers to develop complementary strategies, such as liposome-based approaches, to mitigate the associated side effects [106]. Although hyperoxia does not directly abrogate the tumor cell growth, its effect on the infiltration of antibodies directed to solid tumors without affecting other organs has led to efforts to harness this therapy.

The use of hyperbaric therapy is not without its disadvantages. Recovery of oxygen levels in defined tissues seems to be transient, requiring multiple sessions of hyperoxic breathing to ensure the anti-tumor effect mediated by the immune system [107,108]. Furthermore, various side effects are associated with hyperbaric therapy, including oxygen poisoning, cardiotoxicity, asthma, and barotrauma. Thus, personalized medicine must assess the advantages and disadvantages to determine its usefulness for cancer patients.

Therefore, understanding the influence of oxygen concentration on the secretion of metabolites in cancer cells and its impact on the immune system is crucial for developing new experimental approaches to personalized therapy (Fig. 4B). In this context, a combination of immunotherapy with antagonists of the adenosinergic pathway or lactate production, as well as hyperbaric oxygen therapy could be beneficial for preventing cancer progression and tumor relapse. The use of individual or combined strategies based on the modulation of lactate, adenosine, and oxygen within the TME is currently in clinical trials to overcome hypoxic solid tumors, as listed in Table 1.

**Table 1:** Clinical strategies targeting regulators of adenosine, lactate and oxygen

Treatment	Target	Type of cancer	Observation	Reference
Oleclumab	CD73	Non-small cell lung cancer	Combined treatment with osimertinib increased the progression-free survival of patients.	[109]
Oleclumab	CD73	Colorectal cancer, pancreatic adenocarcinoma, lung cancer	Increased anti-tumor activity and decreased CD73 enzymatic activity.	[110]
MEDI9447	CD73	Colorectal cancer	Increased effector CD8 population and decreased tumor cell growth in mice.	[111]
CD73-specific siRNA-loaded nanoparticles	CD73	Mammary carcinoma	Decreased cell proliferation and sustained tumor regression, lymphangiogenesis in the tumor site and survival of mice.	[112]
Dalutrafusp	CD73-TGF-β	Solid tumors	Decreased soluble CD73 and TGF-β.	[113]
AB598	CD39	Myeloma tumor cells	Increased anti-tumoral immunity and decreased tumor cell growth in mice.	[114]
TTX030	CD39	Melanoma cells	Increased specificity to inhibit CD39 and ADP hydrolysis.	[115]
MSLN-CAR T-cells secreting anti-CD39	CD39	Ovarian cancer cells	Decreased tumor cell growth and increased efficacy of immunotherapy.	[116]
5-aminolevulinic acid (5-ALA)	LDH	Glioblastoma	Increased cell death in cells dependent on glycolysis.	[117]
12-O-deacetyl-phomoxanthone A (12-ODPXA)	PDK4	Ovarian cancer	Reduced tumor growth and migration *in vivo*. Suppressed glucose consumption, lactate secretion, and intracellular ATP production.	[118]
Sononeoperfusion	Oxygenation	Colon cancer and lung carcinoma	Decreased immunosuppressive TME and increased tumor-infiltrating cytotoxic lymphocytes. Combined treatment with anti-PD-LA enhanced tumor regression and prolonged survival.	[119]
Cryptotanshinone (KIS37)	PDK4	Pancreatic cancer	Decreased anchorage-independent growth, CSC markers and colony formation. Suppressed cell growth in the orthotopic tumor model.	[120]
Modified nanoparticles with metabolic inhibitors	Lactate and 6-phosphofructo-2-kinase (PFK-2)	Melanoma	Increased immunocompetent TME. Combined treatment with anti-PDL1 decreased tumor growth.	[121]
PGL13, PGL14, NHI-1 and NHI-2 inhibitors	GLUT-1 and LDH-A	Malignant mesothelioma	Decreased cell proliferation and cell survival, with a higher effect in combined treatment	[122]
Metformin	MCT1 and MCT4	Chronic myelogenous leukemia	Decreased cell proliferation.	[123]
A2aR-specific siRNA-loaded polyethylene glycol (PEG)-chitosan-lactate nanoparticles	A2aR	Breast cancer	Combined with the Dendritic Cell vaccine, it induced tumor regression and increased survival. Also, combined treatment decreased immunosuppressive cells, angiogenesis and metastasis	[124]
FX11 and AR-C155858	LDHA and MCT1, respectively	Breast and colorectal cancer	Combined treatment of AR-C155858 and FX-11 decreased proliferation under normoxia or hypoxia	[125]
Hyperbaric Oxygen Therapy (HBOT)	O_2_	Breast cancer	Reduction of tumor cell growth in combination with conventional therapy	[126]
HBOT	O_2_	Breast cancer	Decreased fibrosis associated with cancer disease, without reducing the pain of patients.	[127]
HBOT + anti-PD1	O_2_	Different types of cell lines	Enhanced the T-lymphocytes infiltration in TME, decreased the extracellular matrix accumulation, and decreased the tumor cell growth	[105]
CAR T-cells + Adenosine deaminase	CD19/HER2	Different types of solid tumors	Enhanced effector activity of CAR T-cells on their target cells in cell lines and mice	[128,129]

## Future Perspectives

Enormous progress has been made in describing the metabolic patterns of cancer cells, and it has been demonstrated that non-cancerous cells also undergo metabolic reprogramming through the interplay of extracellular metabolites. This evidence may suggest that research has reached its limits, but it is only the “tip of the iceberg” in a field of knowledge that must be applied to cancer patients. What next? Firstly, research focused predominantly on the HIF-1α subunit; however, further evidence initiates to recognize that HIF-2α and HIF-3α may have an important role in tumor progression. Since the functions of HIF-1α are not limited to a hypoxic microenvironment, a similar aspect may apply to HIF-2α and HIF-3α subunits. Furthermore, the specific use and design of HIF-α inhibitors and their systemic side effects must be carefully addressed.

Research on multiple metabolites is less explored, but a master transcriptional response associated with hypoxia may aid in explaining the extracellular accumulation of both lactate and adenosine. Thus, hypoxia serves as a common driver of their accumulation, making HIF blockade or oxygen recovery a potentially feasible therapeutic strategy in solid tumors. Indeed, a therapeutic strategy that restricts adenosine and lactate is proposed. Understanding the relationship between these metabolites and the immune system, immunotherapy in combination with antagonists of the adenosinergic pathway or lactate production, and hyperbaric oxygen therapy, could be beneficial in preventing cancer progression and tumor relapse. However, clinical trials once again demonstrate that it is necessary to delve deeper into the complexities of a physiological state. Further studies evaluating the synergistic effect of both metabolites on different hallmarks of cancer, including evasion of the immune system, will allow us to take a key step toward harnessing this knowledge for the benefit of patients.

## Conclusions

Hypoxia is a hallmark of solid tumors characterized by the abundance of extracellular lactate and adenosine. Since oxygen is closely linked to cell metabolism, the effect of HIFs on metabolic reprogramming has predictably been widely discussed [130,131]. A compartmentalized metabolic signature in TME exists, so rather than waste, lactate, and adenosine are master regulators of immune restriction. Advances have addressed additional areas such as the immunological field, suggesting that a balance allows tumor progression [132,133]. The subsequent agreement would undoubtedly require a dialogue between cancerous and non-cancerous cells, within which the metabolites act as messages traveling in both directions.

The scarcity of oxygen, the elevation of adenosine and lactate, as well as the extracellular acidosis, remarkably compromise the immune response [29]. For lactate, the expression of defined MCT1/4 transporters in cancer and immune cells allows for explaining the effect on TME, since for their uptake or release. The alteration of immune cells under hypoxia has been widely addressed [4] and the dual functions of extracellular lactate and adenosine influence the macrophages’ polarization, the cytolysis of NK and T-lymphocytes, or the T-reg cell infiltration. Thus, the immune cell infiltration of hypoxic tumors is entirely modified by extracellular metabolites.

The use of metabolic disruptors or adenosinergic antagonists can be beneficial in enhancing the immunotherapy effect. Although *in vitro* and *in vivo* assays sustain this premise, their usefulness in cancer patients needs to be carefully explored. The plasticity of the metabolism in the face of a constantly changing environment explains the difficulty of a comprehensive view, but understanding how metabolites, adenosine, and lactate, in a hypoxic context, alter the immunological state brings us closer to identifying therapeutic hotspots.

## Data Availability

Data sharing is not applicable to this article as no datasets were generated or analyzed during the current study.
